# Liver-derived metabolites as signaling molecules in fatty liver disease

**DOI:** 10.1007/s00018-022-04658-8

**Published:** 2022-12-07

**Authors:** Umur Keles, Jin Rong Ow, Katharina Barbara Kuentzel, Li Na Zhao, Philipp Kaldis

**Affiliations:** 1 Department of Clinical Sciences, Clinical Research Centre (CRC), Lund University, Box 50332, 202 13 Malmö, Sweden; 2 Institute of Molecular and Cell Biology (IMCB), A*STAR (Agency for Science, Technology and Research), 61 Biopolis Drive, Proteos, Singapore, 138673 Republic of Singapore; 3 Lund University Diabetes Centre (LUDC), Clinical Research Centre (CRC), Lund University, Box 50332, 202 13 Malmö, Sweden

**Keywords:** Liver, Fatty liver disease, Metabolomics, Free fatty acids, Bile acids, Uric acid, Hepatokines, Organokines, Metabolites, Interorgan crosstalk, NAFLD, NASH

## Abstract

Excessive fat accumulation in the liver has become a major health threat worldwide. Unresolved fat deposition in the liver can go undetected until it develops into fatty liver disease, followed by steatohepatitis, fibrosis, cirrhosis, and eventually hepatocellular carcinoma. Lipid deposition in the liver is governed by complex communication, primarily between metabolic organs. This can be mediated by hormones, organokines, and also, as has been more recently discovered, metabolites. Although how metabolites from peripheral organs affect the liver is well documented, the effect of metabolic players released from the liver during the development of fatty liver disease or associated comorbidities needs further attention. Here we focus on interorgan crosstalk based on metabolites released from the liver and how these molecules act as signaling molecules in peripheral tissues. Due to the liver’s specific role, we are covering lipid and bile mechanism-derived metabolites. We also discuss the high sucrose intake associated with uric acid release from the liver. Excessive fat deposition in the liver during fatty liver disease development reflects disrupted metabolic processes. As a response, the liver secretes a variety of signaling molecules as well as metabolites which act as a footprint of the metabolic disruption. In the coming years, the reciprocal exchange of metabolites between the liver and other metabolic organs will gain further importance and will help to better understand the development of fatty liver disease and associated diseases.

## Introduction

Hepatic fat accumulation, also known as fatty liver disease, is a rising global health problem affecting more than 25% of adults worldwide [1, 2] and creates a substantial burden for our society. The incidence of fatty liver disease will probably increase in the following years if the current trends continue [3]. Fatty liver disease is strongly associated with comorbidities such as obesity, type 2 diabetes (T2D), hyperlipidemia, hypertension, and the metabolic syndrome [2]. Although weight loss improves liver histology and thus has a positive effect on disease progression, patient compliance with strict diets is usually low, with a high relapse rate [4]. Therefore, a better understanding of the molecular mechanisms causing the disease and new targets for therapeutic intervention are urgently needed.

Although the accumulation of lipid droplets (LDs) in hepatocytes has long been recognized as the hallmark of fatty liver disease, it is now widely accepted that the storage of excess lipid molecules in the form of triacylglycerol (TAG) is a protective mechanism against cellular lipotoxicity [5–7]. Lipid accumulation is probably a result of overflow after the adipose tissue expansion is exceeded. Indeed, hepatocytes undergo cellular stress due to lipotoxicity when the excess accumulation of free fatty acids (FAs) cannot be disarmed through the lipid storage mechanism or FA oxidation [8–10]. Untreated fatty liver disease-induced stress can aggravate and lead to hepatocyte injury and eventually cell death, the hallmark of non-alcoholic steatohepatitis [11]. Further progression of steatohepatitis may lead to fibrosis, cirrhosis, and eventually hepatocellular carcinoma, the fourth leading cause of death from cancer worldwide [12]. Recently a group of experts agreed on revising and updating the nomenclature and disease definition for non-alcoholic fatty liver disease (NAFLD) and NASH to metabolic-associated fatty liver disease (MAFLD) [13]. This new definition better reflects the complexity of the disease and its consequences and integrates metabolic dysfunction into the terminology. However, to keep it consistent and easier to follow, we will use the term fatty liver disease throughout the manuscript.

Fatty liver disease is considered a liver-focused disease. However, its strong association with obesity, T2D, and the metabolic syndrome suggests a complex metabolic network between liver and adipose tissue as well as other tissues (e.g., pancreatic islets, muscle, heart, and others). Therefore, we need to consider “interorgan crosstalk”, which can be defined as signaling between different tissues promoted by secreting factors into the bloodstream. The importance of fatty liver disease-associated interorgan crosstalk affecting peripheral tissues via hormones, organokines [14, 15], microRNAs [16], and extracellular vesicles [17] has been well recognized. However, how energy homeostasis byproducts secreted from the liver during the development of fatty liver disease and steatohepatitis orchestrate other metabolic players such as muscle, adipose tissue, and pancreas requires further study. Being the master regulator of lipid metabolism, the liver robustly responds to metabolic dysregulation by fine-tuning the lipid output. Another liver-specific function is the synthesis, secretion, and absorption of bile acids. Bile acids in the plasma act as signaling molecules and are altered in many liver diseases. Therefore, the role of bile acids in fatty liver disease development will also be covered in this review. Finally, the liver is exposed to large amounts of dietary fructose due to unhealthy eating habits and the liver’s coping mechanism is closely associated with uric acid metabolism. Thus, in this review, we intend to focus on how liver-derived lipids, bile acids, and uric acid, are involved in interorgan crosstalk that influences energy homeostasis in the context of the development of fatty liver disease.

## Liver-derived lipids as signaling molecules

**Fig. 1:**
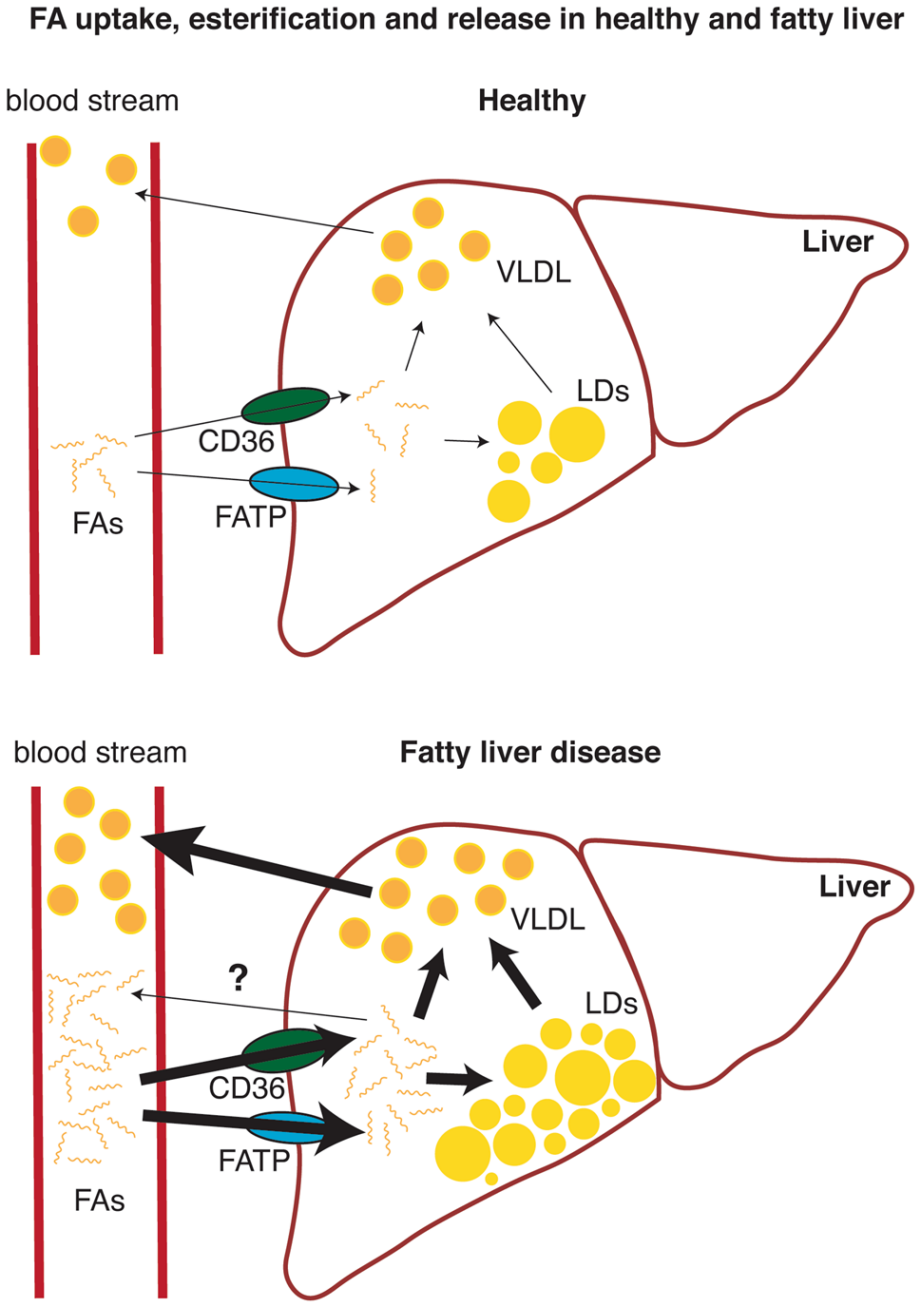
Under physiological conditions, free fatty acids (FA) taken up from plasma by the liver through specific CD36 and FA transport protein (FATP) family receptors can undergo β-oxidation (not shown), Lipid droplet (LD) formation or VLDL formation, depending on the energy needs of the body. However, the metabolic conditions leading to elevated plasma FA concentrations result in increased FA uptake, leading to elevated intracellular FA to which the liver responds by increasing LD deposition, β-oxidation, and VLDL formation. Current evidence also indicates that FAs might be released or transported to the bloodstream as a response to intracellular FA increase

The human body is evolutionarily well-adapted to control energy homeostasis by storing excess energy in the form of neutral lipids that can be used when nutrients are scarce. Maintaining energy homeostasis is demanding and requires robust orchestration between various tissues. Undoubtedly, the liver is a master regulator of this metabolic network, as it regulates a variety of metabolic functions, including but not limited to the regulation of glucose synthesis (i.e., gluconeogenesis), glycogen storage, and bile acid synthesis. The liver also fine-tunes lipid distribution in the body by plasma lipid uptake, de novo lipid synthesis, and lipid secretion into the bloodstream. Of course, adipose tissue is the main lipid storage of the body and the primary source of plasma FAs. Upon high energy demand (fasting or exercise), adipocyte lipolysis releases FAs from TAGs into the bloodstream for transport to the liver and other organs as an additional source of energy [18]. Hepatocytes then take up plasma FAs either by FA transport proteins (FATP) 2, 5 [19], the scavenger receptor CD36 [20, 21] or to a lesser extent passive diffusion [22]. Moreover, FAs are also synthesized in the liver or taken up from the diet in the form of chylomicron remnants. In the case of obesity-induced insulin resistance, increased adipocyte lipolysis releases more FAs from the TAG stores into circulation [23]. Ironically, this might occur due to endoplasmic reticulum (ER) stress caused by increased dietary FA flux [24] and excessive expansion of adipocytes leading to adipose insulin resistance [25]. Therefore, the net outcome is increased levels of serum FA which are taken up by the liver. The resulting accumulation of FAs in hepatocytes is either used in mitochondria for β-oxidation or esterified into TAG. Hepatic accumulation of TAGs is either utilized to form very low-density lipoproteins (VLDL) or stored in LDs in hepatocytes, the phenotypic trademark of fatty liver disease (Fig. 1). If the elevated FA influx cannot be compensated by intracellular coping mechanisms, further FA accumulation results in lipotoxicity, which eventually leads to cellular damage.

**Fig. 2:**
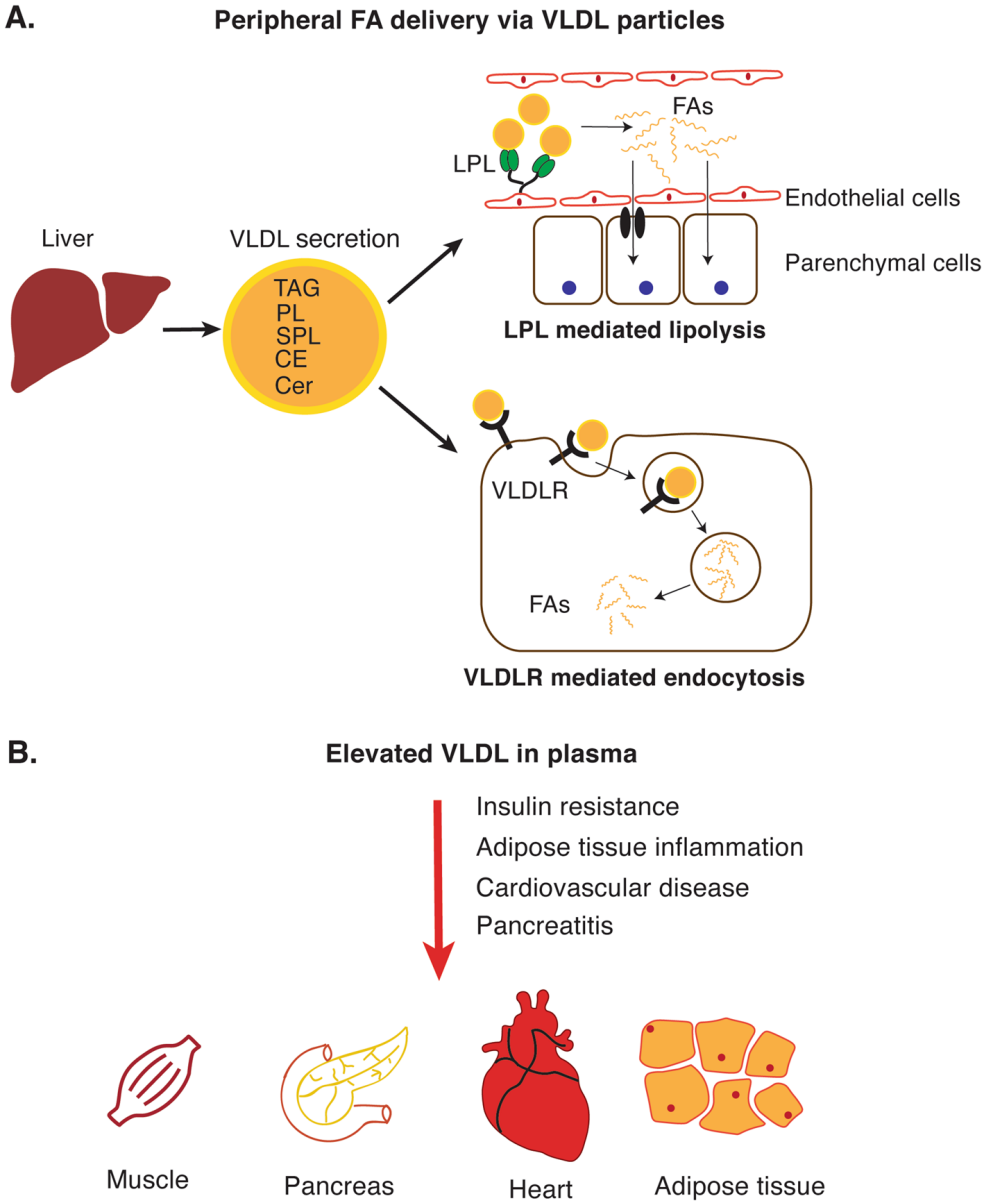
**A** TAG molecules constituting the VLDL particles secreted from the liver can be liberated by lipoprotein lipase (LPL). Free fatty acids (FAs) can then be taken up by CD36 or FA transport protein receptors. Alternatively, FAs can pass through the lipid bilayer by passive diffusion. VLDL particles can also be taken up directly by target tissues via VLDL receptor (VLDLR)-mediated endocytosis. **B** The amount and the composition of VLDL is altered during the development of fatty liver disease. While LPL-mediated FA uptake is increased in adipose, muscle and heart tissue; receptor-mediated VLDLR uptake is critical for the pancreas. The red arrow indicates the deleterious effect of increased VLDL concentrations. *TAG* triacylglycerol, *PL* phospholipids, *SPL* sphingolipids, *CE* cholesteryl esters, *Cer* ceramide

One of the liver’s coping mechanisms for elevated intracellular lipid concentrations is to secrete them in VLDL particles, rich in TAG and cholesteryl esters [26]. VLDL delivers energy-rich TAG to peripheral tissues via the bloodstream, where in endothelial cells lipoprotein lipase (LPL) hydrolyzes TAG to liberate diacylglycerol (DAG) and FA [27]. Adipose tissue, heart, and skeletal muscle express high levels of LPL and are exposed to TAG-derived metabolites. Peripheral tissues take up released FA via FA transporter receptors including CD36 and FATP [28] or passive diffusion (Fig. 2A).

During the development of fatty liver-associated comorbidities such as T2D and obesity, the liver secretes large amounts of VLDL particles [29] that eventually lead to elevated levels of plasma FA uptake through lipase activity (LPL) [30]. Indeed, increased FA uptake largely contributes to tissue insulin resistance, which was shown by muscle-specific deletion of LPL [31]. Lipotoxicity-induced cardiomyopathies are among fatty liver disease-, T2D-, and obesity-associated comorbidities [32, 33]. FAs are the primary energy source of cardiomyocytes and can either be taken up directly from plasma or from lipoproteins via LPL. However, lipoprotein-derived FAs seem to be a limiting factor for cardiac lipid uptake as heart-specific LPL deletion increases plasma TGs but not plasma FAs [34].

The role of the adipose tissue in increasing plasma FAs, thereby increasing liver lipid deposition in the context of obesity and T2D is well acknowledged and has been extensively discussed elsewhere [35, 36]. During fatty liver disease development, the liver also influences the remodeling of the adipose tissue. VLDL, for example, is known to be a major source of lipids for the adipose tissue since the deletion of the VLDL receptor (VLDLR) leads to decreased adipogenesis in high-fat diet-fed mice [37]. Although adipose VLDLR is decreased in morbidly obese patients [38], it mediates excessive VLDL uptake in macrophages and exacerbates adipose tissue inflammation [39, 40].

Although LPL expression in the pancreas is relatively low, evidence indicates that elevated VLDL levels contribute to the development of pancreatitis, as a common condition among obese and T2D patients [41, 42], and are further known to be associated with fatty liver disease [43]. In mice, deletion of the VLDLR in pancreatic stellate cells ameliorates the development of pancreatitis [44].

Ceramides belong to a class of lipids called sphingolipids, essential components of cellular membranes [45] and ceramide synthesis favors saturated long-chain FA over unsaturated or short-chain FA [46]. Ceramides produced in the liver are distributed to the other organs through VLDL and plasma ceramide levels are strong predictors of insulin resistance and cardiovascular diseases [47–49]. Interestingly, unlike in the liver, ceramide levels are associated with insulin resistance in skeletal muscle [50, 51]. Studies have shown that ceramides negatively influence the AKT pathway [52–54], the primary signal transduction mode of the insulin receptor. Studies suggest that this might happen by preventing AKT from translocating to the plasma membrane [55] through PKCζ-mediated inhibition of phosphatidylinositol 3,4,5-trisphosphate binding the AKT pleckstrin homology domain [56, 57] or by augmenting protein phosphatase 2A (PP2A) dephosphorylation of AKT residues [57, 58]. Ceramides may also impair the insulin response in skeletal muscles independent of AKT by inhibiting glucose transporter 4 (GLUT4) translocation to the plasma membrane, thereby reducing muscle glucose uptake [59]. Excess circulating glucose upon skeletal muscle insulin resistance might be taken up by the liver and serves as a source for de novo lipogenesis, of which the primary output is saturated long-chain FA, culminating in further lipid deposition in the liver and secretion in VLDL particles [60]. Recent studies revealed that senescent hepatocytes and adipocytes can promote the development of metabolic diseases. This occurs either by reducing mitochondrial FA oxidation, causing hepatic lipid accumulation and fatty liver disease [61] or by triggering the NFkB-dependent senescence-associated secretory phenotype in adipocytes and causing adipose inflammation [62], a hallmark of diabetes. Ceramides have been shown to promote senescence [63] through PP1 and PP2A-mediated dephosphorylation and inactivation of cyclin-dependent kinase 2 (CDK2) [64]. Taken together, it is not surprising that blocking ceramide synthesis is effective in preventing diet-induced insulin resistance [65]. Conversely, senotherapeutic drugs (inhibiting senescence) may be useful too, since they have recently been shown to improve the regenerative capacity of the adult liver [66]. In summary, these new types of drugs suggest a bright future for treating liver diseases or at least some of their clinical manifestations.

FA are the building blocks of membranes, stored lipids and efficient energy substrates, composed of hydrocarbon chains linked to a carboxylic acid group. FAs that are taken up by the cell, are either re-esterified or activate peroxisome proliferator-activated receptor α (PPARα) to induce mitochondrial and peroxisomal β-oxidation to yield acetyl-CoA [67, 68], with the ultimate goal of generating ATP. Accordingly, PPARα levels are increased in fatty liver disease patients and its deletion leads to the worsening of steatosis, reflecting a compensatory mechanism against excessive FA influx [69]. However, NASH patients exhibit reduced PPARα levels [70] which further exacerbates the condition by impairing FA clearance and causing more FAs to enter the TAG synthesis pathway in a vicious cycle. Overload of cellular FA exceeding the capacity of TAG synthesis leads to increased DAG levels, an intermediate metabolite, which activates protein kinase C (PKC) and blunts insulin receptor signaling [71], resulting in hepatic insulin resistance. It is thus not a coincidence that about 34–70% of diabetic patients also suffer from fatty liver disease [72].

The type of circulating FAs can impact other organs besides the liver. Medium- and long-chain FA are ligands for the receptor GPR40 and act on pancreatic β-cells to enhance the secretion of insulin [73] by inducing the endoplasmic reticulum (ER) to release its calcium stores and triggering exocytosis of insulin vesicles [74]. In addition to the pancreas, FAs activate GPR40 as well as GPR120 on the enteroendocrine cells of the gut to induce the secretion of incretins and indirectly promote pancreatic insulin secretion [75, 76]. Notably, we have observed that the chronic release of excessive amounts of FA into the blood can lead to insulin resistance by sustaining elevated blood insulin levels in a mouse model with dysfunctional hepatic mitochondrial β-oxidation [77]. This FA-mediated organ crosstalk might provide a mechanism by which organ-specific insulin resistance can develop into systemic insulin resistance. Interestingly, GPR40 deficiency protects from insulin resistance [78] while GPR120 deficiency results in obesity and insulin resistance [79]. This discrepancy might be due to a preference for GPR40 for saturated FA and GPR120 for unsaturated FA [80], suggesting a difference in metabolic regulation by saturated and unsaturated FA [81].

Short-, medium- and long-chain FA, can influence insulin secretion indirectly. Short-chain FAs (SCFAs) are ligands of GPR41 and GPR43 and promote the secretion of incretins by enteroendocrine cells of the gut [82]. Paradoxically, SCFAs inhibit insulin signaling only in adipocytes through the activity of GPR43 but increase insulin sensitivity in other organs. This is proposed to prevent excessive storage of energy in the adipose tissue while enhancing energy expenditure in other organs. Of note, this effect was dependent on the composition of the gut microbiota, suggesting microbes are the primary sources of SCFAs [83, 84]. This indicates the importance of metabolite signaling between the gut microbiota and the metabolic organs of our body and will be discussed further below in the context of bile acids. The human body produces SCFAs as a product of β-oxidation and ketogenesis or from ethanol by mitochondrial acetyl-CoA hydrolase (ACOT9) upon ethanol consumption. Free acetate, a type of SCFA, is not used by the liver for ATP generation since hepatic acetyl CoA synthetase has a low affinity for acetate [85]. Acetate is more likely delivered to the heart, as the cardiac acetyl-CoA synthetase has a high affinity for acetate and converts it to acetyl-CoA for energy [85]. On the other hand, excessive ethanol consumption leading to elevated levels of plasma acetate and, correspondingly, acetyl-CoA, can enhance histone acetylation in immune cells to up-regulate the expression of inflammatory cytokines, contributing to acute alcoholic hepatitis [86] or in hippocampal cells to modulate transcriptional responses of alcohol-associated learning and memory [87].

As discussed above, FAs are primarily released from the adipose tissue. Indeed, increased plasma FAs, as in obesity, are scavenged by the liver, leading to elevated lipid deposition. Excessive lipid deposition, namely liver steatosis, triggers compensatory mechanisms by enhancing VLDL formation and secretion, thereby reducing the lipid burden of the liver [26]. Although VLDL secretion is best known as a FA export mechanism, a recent study on liver-specific CDK mutant mice has shown that the liver might directly release FAs into the plasma [77]. CDK family proteins are master regulators of the cell cycle [88]. Among other CDKs, CDK1 drives the cell cycle through G2 to mitosis when bound by the cyclin B1 protein; and its deletion results in mitosis failure, but hepatocytes grow further in size [89]. We have shown that *Cdk1* deletion in hepatocytes, thereby preventing mitosis, results in the depletion of liver TAGs and substantially decreased plasma VLDL levels. Partially due to elevated expression of liver adipose triglyceride lipase (ATGL, encoded by *PNPLA2*), the rate-limiting enzyme releasing FA from cytosolic LDs [90]. Surprisingly, however, plasma FA concentrations were significantly elevated in liver-specific *Cdk1* mutant mice [77]. As a result, FAs affected the physiology of the adipose tissue, muscle, and pancreatic islets, resulting in hyperinsulinemia. Over time, these animals developed insulin resistance, and the increased blood glucose levels activated the transcription factor liver X receptor (LXR). The LXR/RXR dimer then promotes the expression of lipogenic genes, resulting in liver steatosis. Interestingly, the phenotype of this mouse model with a mutation in hepatocytes was dependent on interorgan crosstalk, suggesting that more than one organ is at fault in T2D and fatty liver disease [77].

Although the fatty liver disease-associated mechanisms seem to contribute to the total lipid burden of the body (Fig. 2B), there is still a need for a deeper understanding of how the liver remodels peripheral tissues during the development of the disease. Future studies on diverse lipid species secreted from the liver and their effects on target tissues would pave the way to develop more precise therapeutic molecules against fatty liver disease or associated diseases.

## Bile acids

In addition to regulating metabolic functions, the liver also plays a critical role, together with the digestive system, to control bile acid production, secretion, and recycling. Although bile acids are well-acknowledged for their function in emulsifying lipids in the intestinal lumen, it has also been shown that plasma bile acid concentrations are dysregulated during the development of fatty liver disease [91] and more importantly, they act as signaling molecules on peripheral tissues [92].

Primary bile acids produced by hepatocytes from cholesterol are chenodeoxycholic acid (CDCA) and cholic acid (CA). These are typically conjugated to glycine or, to a lesser extent, taurine before secretion [93]. Conjugated bile acids are usually referred to as bile salts, stored in the gallbladder and secreted via the bile duct into the duodenum, which is mediated by the ATP-binding cassette subfamily B member 11 (ABCB11) and multidrug resistance-associated protein 2 (MRP2) transporters [94]. Up to 95% of primary bile acids are recycled in the ileum, either via the apical sodium-dependent bile acid transporter (ABST) found on enterocytes or via passive diffusion [95, 96]. Enterocytes secrete bile acids into the portal circulation via the organic solute transporter α (OSTα) and OSTβ proteins [97]. Portal bile acids are then actively transported to hepatocytes by the organic anion transporting polypeptide (OATP) and sodium taurocholate co-transporting polypeptide (NTCP) [98]. A portion of the bile salts, however, becomes deconjugated by microbial hydrolases, and bacterial enzymes further convert these into secondary bile acids: lithocholic acid (LCA) and deoxycholic acid (DCA), which are derived from CDCA and from CA [99], respectively. Therefore, the gut microbiome plays an important role in the recycling of bile acids. Secondary bile acids can then re-enter the enterohepatic system by passive diffusion and serve as signaling molecules [100]. A small portion of bile acids in the portal circulation may escape liver uptake and can be found in the bloodstream. Not surprisingly, serum bile acids are elevated postprandially [101]. However, the liver robustly clears the remaining bile acids from circulation. Therefore, the persistency of high bile acid concentration in the circulation has been associated with liver diseases [102–104] and can be manifested by jaundice.

The presence of circulating bile acids is primarily recognized by two receptors found in peripheral tissues: the farnesoid X receptor (FXR) and G Protein-Coupled Bile Acid Receptor 1 (TGR5). Unconjugated primary bile acids are taken up through the intestines, bind, and activate the transcription factor FXR [105, 106]. This allows for negative feedback on bile acid synthesis on two fronts. Activation of hepatic FXR promotes expression of nuclear receptor subfamily 0 group B member 2 (NR0B2), which binds and represses liver receptor homolog-1 (LRH-1) transcriptional activity, thereby reducing the expression of cholesterol 7 alpha-hydroxylase (*CYP7A1)*, a key enzyme in bile acid synthesis [107, 108]. Alternatively, activation of FXR in the intestine induces fibroblast growth factor 15 (FGF15) production, which travels to the liver via the portal vein and stimulates the FGFR4-JNK-NR0B2 pathway in hepatocytes to inhibit the transcription of *CYP7A1* [109]. Primary bile acids also regulate liver lipid metabolism by repressing the transcription factor PPARγ coactivator 1- α (PGC-1α) using the FXR-NR0B2 axis [110], especially given the crucial role of PGC-1α in FA oxidation and utilization [111].

**Fig. 3:**
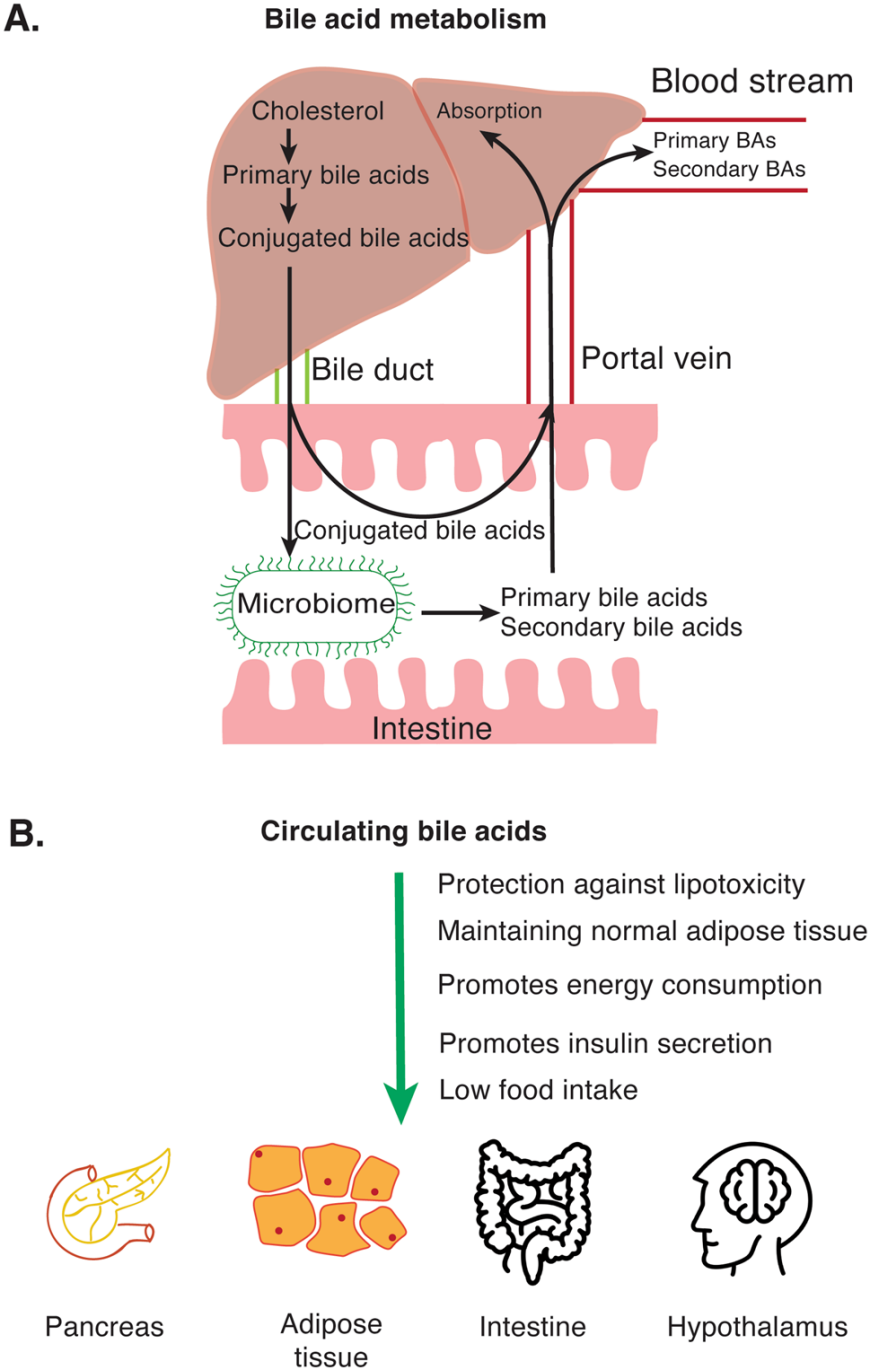
**A** The liver synthesizes primary bile acids from cholesterol. Bile acids are also conjugated either with glycine and taurine and become conjugated bile acids (CBAs). CBAs are secreted into the intestines through bile ducts. CBAs can be deconjugated and/or turned into secondary bile acids by the microbiome. Primary and secondary bile acids are then absorbed through the ileum and shuttled back to the liver through the portal vein. Although most of the bile acids are taken up by hepatocytes, a small portion escapes from absorption and is released into the bloodstream. Bile acids can directly bind to the nuclear receptor Farnesoid X receptor (FXR) to mediate gene regulation or act on G-protein coupled receptor TGR5 to initiate an intracellular signal cascade. **B** Although plasma bile acids seem to increase in patients with fatty liver, the effect on peripheral tissue is mostly determined as anti-diabetic (green arrow)

The in vivo data reveals a tissue-specific impact of FXR function on the metabolic outcome. Whole-body FXR-deficiency in mouse models of insulin resistance protects mice from diet-induced obesity and glucose intolerance [112–114], independent of hepatic FXR, as hepatic insulin resistance remains unaffected in the absence of FXR [112]. In this context, intestinal FXR appears to be responsible for promoting systemic insulin resistance, as intestine-specific inhibition of FXR led to a reduced ceramide synthesis and therefore prevented and reversed the development of fatty liver disease [115], possibly by preventing adipose inflammation caused by bile acids produced by the gut microbiota [114]. In contrast, hepatic FXR is protective against fatty liver disease, as liver-specific knockout of *Fxr* enhanced the incidence of hepatic steatosis [116]. A recent study supports the tissue-specific responses of FXR activation by providing evidence that hepatic FXR modulates lipogenesis, whereas intestinal FXR controls lipid absorption from the gut [117]. Nevertheless, conjugated bile acids might be antagonists of FXR [118, 119], instead of unconjugated bile acids. Since bile acids are released from hepatocytes in the conjugated form and 95% of the conjugated bile acids are absorbed from the ileum [120] (Fig. 3A), hepatic FXR exposed to the extracellular environment in the liver are more likely to encounter conjugated bile acids and become inhibited. On the other hand, intestinal cells are more likely to be exposed to unconjugated bile salts as the gut microbiota deconjugates a small percentage of bile acids that are released into the intestines to promote FXR signaling [119].

Besides the entero-hepatic system, although expressed at relatively low levels, FXR activation in various tissues significantly impacts energy homeostasis. In the pancreas of obese animals, FXR translocates to the nucleus and protects pancreatic islet cells against lipotoxicity [121]. In another study, FXR activation in β-cells has been shown to stimulate insulin secretion and glucose uptake [122]. Another strong implication of the roles of FXR in energy metabolism is adipose tissue: animals lacking FXR show impaired adipose tissue development and insulin resistance [123]. Interestingly, hypothalamic FXR has been reported to have a residual expression profile [124] but modulates brown adipose tissue through the sympathetic system upon FXR agonist treatment [125].

Obviously, findings of FXR activity are contradictory since fatty liver disease patients show elevated levels of serum bile acids despite the bile acid receptor FXR being mostly associated with improved energy homeostasis [126]. This contradiction could partially be attributed to the bile acid composition instead of the total amount of bile acids. In one clinical study, although serum total bile acid concentration was significantly elevated in fatty liver disease patients compared to the healthy cohort, the amount was not significantly altered between patients suffering from fatty liver or steatohepatitis [127]. Conversely, conjugated bile acid ratios were decreased in NASH compared to fatty liver disease [127] while cholic acid conjugates are increased in NASH. The other plausible explanation could be the differential effect of bile acids on FXR receptors. One such example is that 12α-hydroxylated secondary bile acids, LCA and DCA, increase during T2D and are known to antagonize FXR [128].

Secondary bile acids like taurolithocholic acid (TLCA), LCA, and DCA [129], produced by the gut microbiome from primary bile acids, are preferential ligands for the receptor TGR5 [130]. Unlike FXR, studies on TGR5 conclusively point to TGR5 being anti-diabetic. For example, treatment with bile acids activates TGR5 in brown adipose tissues in mice or skeletal muscle in humans to induce type 2 iodothyronine deiodinase activity, which increases the active thyroid hormone levels and promotes higher energy consumption [131]. Bile acid-mediated activation of TGR5 also regulates glucose homeostasis by enhancing insulin secretion by pancreatic β-cells through cAMP-PKA signaling [132] or triggering intestinal incretin secretion [133]. Accordingly, TGR5 signaling protects against diet-induced obesity [131, 133] and other diabetes-associated pathologies such as fatty liver disease [134], diabetic retinopathy [135], and nephropathy [136]. Notably, observations in humans subjected to CDCA treatment support the findings in vitro and in mice, with elevated brown adipose tissue activity and energy expenditure upon administration of TGR5 agonists but not FXR agonists [137]. In distinct tissues such as the nervous system, activation of TGR5 in the hypothalamus was shown to protect against obesity by reducing food intake [138].

The sensors that secondary bile acids can modulate are not restricted to TGR5; they also bind to FXR receptors with high affinity. It is acknowledged that disruption of the gut microbiome abrogates secondary bile acid formation, disrupting TGR5 and FXR stimuli. Of course, this highlights the importance of maintaining a healthy gut microbiome for proper physiological metabolism. Due to the pleiotropic effect of FXR, long-known inhibitors are of limited use in the clinic. Therefore, in addition to other pharmacological interventions for fatty liver disease, the gut microbiome emerges as an alternative therapeutic option.

The recognition of bile acid metabolism and its contribution to energy homeostasis (Fig. 3B) has enabled the discovery of specific bile acid agonists, which are currently under clinical investigation. Although the results are promising, further safety studies are needed for general use in fatty liver disease patients [94]. This clearly indicates that further investigations on how different bile acid species act on peripheral tissues are needed. In addition to chemical intervention, modulation of microbiota would be a promising tool for adjusting specific plasma bile acid species.

## Uric acid

**Fig. 4:**
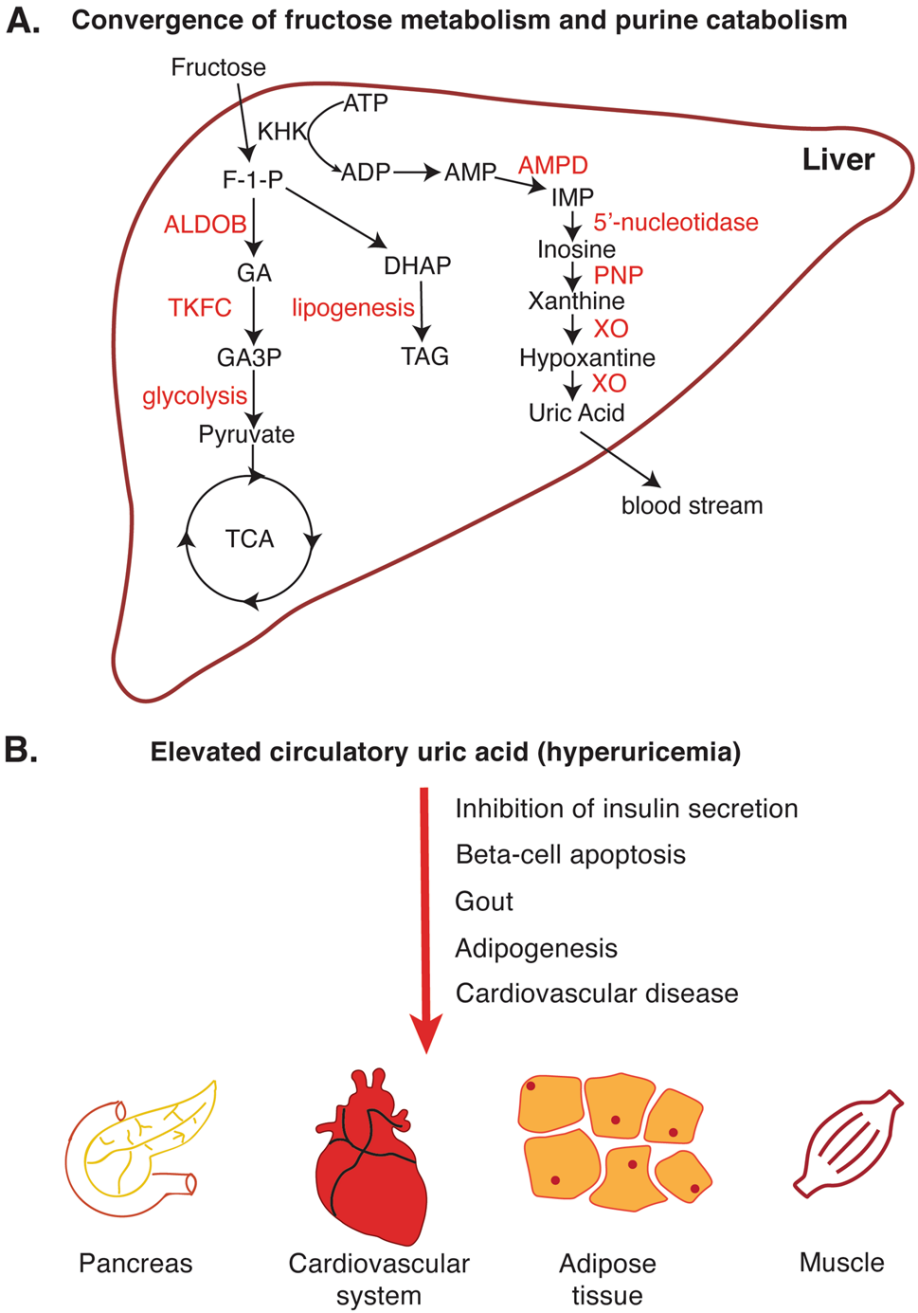
**A** After digestion, the liver is exposed to large amounts of fructose through the portal vein. Excessive fructose leads to a decrease in ATP levels and an increase in AMP concentration and AMP deaminase. Elevated deaminase activity promotes purine catabolism and eventually leads to a net increase in uric acid formation. **B** Elevated uric acid is a well-known cause of gout. It was also shown to inhibit insulin secretion, cause β-cell apoptosis, and adipogenesis. (the red arrow indicates the deleterious effect of hyperuricemia) *TCA* tricarboxylic acid cycle, *F-1-P* Fructose-1-phosphate, *AMPD* AMP deaminase, *PNP* purine nucleotide phosphorylase, *XO* xanthine oxidase, *ALDOB* aldolase B, *TKFC* triokinase/FMN cyclase, *GA* D-glyceraldehyde, *GA3P* D-glyceraldehyde-3-phosphase

Purine molecules, guanine, and adenine mediate critical cellular functions such as monomers in nucleotide synthesis or energy carriers. Purine catabolism takes place in the liver through nucleotidase, deaminase, and xanthine oxidase enzymes (Fig. 4A). The end product of the purine catabolism in the liver is uric acid which is released into the bloodstream and excreted through the kidneys [139]. Disrupted purine metabolism or uric acid excretion leads to elevated serum uric acid levels, namely hyperuricemia. Hyperuricemia has long been associated with gout, the accumulation of urate crystals in joints, causing painful inflammation. Recent studies have also shown that hyperuricemia is also associated with the development of fatty liver disease.

The lifestyle and feeding habits of human beings have dramatically changed over the last decades, mainly due to the industrialization of food production. Similarly, the composition of the diet has also undergone fundamental changes. One such example is high fructose corn syrup which is heavily used in food and beverages as a sweetener [140]. It is well-documented that fructose has the propensity to promote fatty liver disease and other metabolic diseases [141]. Dietary fructose is taken up in the small intestine either by the transporter GLUT5 into intestinal epithelial cells [142], where it is metabolized by the ubiquitous ketohexokinase-C, or by GLUT2 into the hepatic portal system [143] to be delivered to hepatocytes and broken down by the hepatic ketohexokinase-A. The two ketohexokinases are alternatively spliced isoforms that utilize ATP to convert fructose to fructose-1-phosphate, which is converted to glyceraldehyde and dihydroxyacetone phosphate, after which the fructose metabolic pathway converges with glucose metabolism at the glycolytic step (Fig. 4A) [141]. Interestingly, the different routes of fructose uptake have distinct impacts on the development of fatty liver disease. Intestinal uptake and clearance of fructose protect against fructose-induced fatty liver by reducing the amount of fructose, microbiome, and bacterial toxins taken up by the liver, alleviating liver inflammation [144, 145]. On the other hand, hepatic fructose metabolism is linked to hepatic steatosis [141]. High hepatic ketohexokinase activity, a result of excessive fructose consumption overwhelming the intestinal fructose clearance capacity, can cause a drop in ATP and intracellular phosphate levels [146, 147], thereby triggering the activity of AMP deaminase [148, 149], which promotes purine catabolism. This explains the acute rise in serum uric acid, the end product of purine degradation, following fructose consumption [150]. This raises the question: why is uric acid unfavorable for the liver?

The observation that the fructose-mediated metabolic syndrome can be partially rescued by treatment with allopurinol, a xanthine oxidase inhibitor that blocks uric acid production, suggests that uric acid may be a mediator of fructose-dependent fatty liver development [151, 152]. This is supported by studies showing that treatment with xanthine oxidase inhibitors can rescue the steatotic phenotype in fatty liver disease patients and mouse models [153–155]. Notably, increased uric acid levels have been observed in non-dietary rodent models of insulin resistance by others and ourselves [77, 156], and elevated serum uric acid has been identified as a risk factor for the development of fatty liver disease in humans [157–159], implicating a more generic role for purine catabolism in fatty liver disease pathogenesis. Multiple mechanisms for uric acid-dependent fat accumulation in hepatocytes have been suggested. For example, uric acid induces oxidative stress leading to the accumulation of citrate, which serves as raw material for de novo lipogenesis [160], while promoting the expression of lipogenic enzymes via the JNK-sterol regulatory element-binding protein 1-c (SREBP-1c) pathway [161]. Alternatively, uric acid can cause ER stress, thereby enhancing ER stress-dependent cleavage and activation of SREBP-1c [162]. Uric acid may additionally promote insulin resistance by reducing the expression of the organokines FGF21 through the up-regulation of miR-149-5p [163]. FA oxidation is repressed due to redox inactivation of the β-oxidation enzyme enoyl-CoA hydratase 1 [160]. Besides promoting lipid accumulation, uric acid may also facilitate the progression from hepatic steatosis to steatohepatitis by inducing an inflammatory response through the release of C–C chemokine ligand 2 (CCL2) from endothelial cells [164] and activation of the NRLP3 inflammasome [165] via the ROS-TXNIP pathway [166] to promote cell death in hepatocytes [167].

Besides the liver, uric acid can also affect other organs (Fig. 4B). Perhaps, the most studied clinical manifestations of elevated plasma uric acid (hyperuricemia) in peripheral tissues are crystal formation in joints, namely gout [168]. However, hyperuricemia can also affect other metabolic organs including the adipose tissue and pancreas. Uric acid treatment has been shown to inhibit glucose-stimulated insulin secretion in isolated pancreatic islets and pancreatic β-cell lines while inducing β-cell apoptosis [169–171], a common phenomenon seen in T2D. Uric acid can also promote adipogenesis in mesenchymal stem cells, contributing to fructose-mediated obesity [172]. Interestingly, uric acid may be secreted by adipocytes, through enhanced uric acid production by adipose tissues from obese animals [173]. These findings highlight a positive feedback loop between the liver and the adipose tissue and the interplay between purine catabolism and lipogenesis in these two metabolic organs in cases of metabolic disease. In addition to its direct role on metabolic tissues, uric acid has been shown to act on the central nervous system and modulate energy metabolism. In one study, high-uric acid diet fed rats displayed elevated levels of inflammatory cytokines, activated NF-kB pathway and increased gliosis, a reactive response of glial cells, in the hypothalamus. This lead to dyslipidemia and glucose intolerance [174]. Furthermore, uric acid has also been associated with impaired cognitive functions in rat studies [175].

Despite the well-acknowledged anti-oxidant function in plasma, uric acid has also been described as a pro-oxidant in the cytoplasm or in atherosclerotic plaques, thereby causing/accelerating the development of cardiovascular disease [176].

## Conclusions

In the industrialized world, diseases associated with excessive calorie intake have drawn much attention due to chronic but devastating physiological outcomes. In this context, fat accumulation in the liver and the development of fatty liver disease have long been considered associated with obesity and T2D. Here, we discussed selected liver-derived metabolites as a ‘cause’ of direct or indirect actions promoting the development of fatty liver disease.

As one can expect, no single liver-derived molecule has been discovered as the primary contributor to the fatty liver disease itself or the associated diseases. Accordingly, although fatty liver disease biomarkers have been intensively studied, and more metabolites have been covered, the predictive value of the available metabolites is still limited [177]. This is partially due to the current limitation in metabolite coverage of mass spectrometry-based metabolomics [178]. Furthermore, a mere concentration of the plasma metabolites might not reflect true biological functions. For example, plasma lipids derived from the liver are primarily carried in lipoprotein particles [179]. These lipoproteins are able to act as compartments and exchange various lipids (e.g., TAG and cholesteryl ester), which might alter the lipid species delivered via the uptake of lipoproteins in peripheral tissues.

As the number of covered metabolites in untargeted metabolomics studies increases, another issue remains to be tackled: despite primarily being byproducts of physiological processes, many metabolites can enter cells via passive diffusion, while several others are recognized by cellular receptors and taken up by the target tissues. However, many metabolite-specific tissue receptors are yet to be discovered [180].

Indeed, the development of fatty liver disease is a complex process and requires more mechanistic studies to better understand how metabolic disturbances abrogate the lipid compensation mechanisms of the liver. However, it is equally important to elaborate on how the liver responds to the altered physiology.

## Data Availability

This does not apply since this is a review article.
